# Lecithin in Neurasthenia, and in Graves' Disease

**Published:** 1909-01-23

**Authors:** 


### Medicine.

### LECITHIN IN NEURASTHENIA, AND IN GRAVES' DISEASE.

Phosphoric-acid compounds have been known for some time to benefit cases of Graves' disease more or less. More recently Dr. H. J. Berkley has given phosphorus in the organic form of lecithin in these cases. From the published results it seems clear that lecithin does no harm, and there is some evidence to show that the benefits which follow its use in cases of Graves' disease are *propter* and not merely *post*. The drug was employed in the first instance for nervous asthenia (not psychasthenia), and it was partly on account of the good results obtained that Dr. Berkley was led to try it in Graves' disease too.

In the first place lecithin has a nauseous and disagreeable odour and taste. Nothwithstanding this the neurasthenic and goître cases are found to cling to the remedy as an opium habitué does to opium. They never seem to tire of it until the nervous symptoms have been allayed and the weight approaches normal. It is stated that under lecithin treatment these patients experience a sensation of quietude an hour or two after taking each dose, comparable with the tranquillising effect of bromide.

When there is disturbed digestion, however, lecithin should not be given, and in such an event other remedies must be employed to restore the alimentary canal to a healthy state. In some instances, unless a careful watch is kept, an erythematous rash may be produced. Furthermore lecithin without the assistance of a milk diet is an entire failure. It does no good in any case of either nervous asthenia or Graves' disease when milk is not tolerated, and at least one litre of milk must be taken daily by the patient in addition to other food. This being so it might be urged that this dietary might benefit the patients equally well without lecithin; but to test this a number of patients were given during alternate weeks lecithin and a compound preparation of the glycerophosphates of sodium, calcium, iron, and manganese, with or without the glycerophosphate of quinine and extract of gentian. They lost weight and their neurasthenia increased on the glycerophosphates, and gained weight with abatement of the nervous symptoms while on the lecithin.

Only the worst affected were kept absolutely in bed. The average patient was at first kept there from 9 P.M. to 9 A.M., whilst the day was spent out of doors practically idling. After pronounced gain in weight and signs of abatement of the nervous symptoms the lighter portions of the usual daily occupations were begun again; and then, if gain in weight was not interfered with, more and more of the doings of ordinary life were permitted. Dr. Berkley lays stress upon the fact that the treatment of neurasthenic and Graves' disease cases does not cease when lecithin has abated the active symptoms of the complaint. No resource of hygiene should be relaxed. The dietary should remain upon the side of fulness for an indefinite length of time, active exercises should be restricted until the last symptoms of the malady have been gone for some weeks, and it is recommended that small doses of arsenical salts, particularly those of potassium and sodium, should be given in place of the lecithin, to keep up the effects of the latter.

